# Localization and expression profiles of gingival monocyte chemoattractant protein-1-induced protein-1 (MCPIP-1) and mucosa-associated lymphoid tissue lymphoma translocation protein 1 (MALT-1)

**DOI:** 10.1007/s00784-023-05010-5

**Published:** 2023-04-03

**Authors:** Lili Yu, Yigit Firatli, Samira Elmanfi, Mervi Gürsoy, Meltem Özdemir Kabalak, Gökhan Kasnak, Pirkko Pussinen, Floris J. Bikker, Feriha Caglayan, Erhan Firatli, Ulvi Kahraman Gürsoy

**Affiliations:** 1 Department of Periodontology, Institute of Dentistry, University of Turku, Lemminkäisenkatu 2, 20520 Turku, Finland; 2 Department of Periodontology, Faculty of Dentistry, Istanbul University, Istanbul, Turkey; 3 Department of Periodontology, Faculty of Dentistry, Hacettepe University, Ankara, Turkey; 4 Department of Periodontology, Faculty of Dentistry, Istanbul Kent University, Istanbul, Turkey; 5 Department of Oral and Maxillofacial Diseases, University of Helsinki, Helsinki, Finland; 6 Department of Oral Biochemistry, Academic Centre for Dentistry Amsterdam, University of Amsterdam and VU University Amsterdam, Amsterdam, the Netherlands

**Keywords:** MCPIP1 protein, Human/Regnase-1 protein, human, MALT-1 protein, *Porphyromonas gingivalis*, Interleukin-8

## Abstract

**Objectives:**

The purposes of this study were to localize monocyte chemoattractant protein-1-induced protein-1 (MCPIP-1) and its suppressor mucosa-associated lymphoid tissue lymphoma translocation protein 1 (MALT-1) in gingival tissues and to profile their protein expression levels in relation to the clinical inflammation, *Porphyromonas gingivalis* colonization, and interleukin (IL)-8 levels.

**Materials and methods:**

Study samples were collected from two independent study populations: (1) Gingival tissues were collected from eight periodontally healthy individuals and eight periodontitis patients to localize MCPIP-1 and MALT-1 immunohistochemically, and (2) forty-one gingival tissue samples with marginal, mild, or moderate to severe inflammation were collected from 20 periodontitis patients to determine MCPIP-1 and MALT-1 levels using immunoblots, *P. gingivalis* levels with qPCR, *P. gingivalis* gingipain activities with fluorogenic substrates, and IL-8 levels with multiplex technique.

**Results:**

MCPIP-1 was detectable in the epithelium and in connective tissue, being especially prominent around the blood vessel walls in healthy periodontal tissues. MALT-1 was observed at all layers of gingival epithelium and especially around the accumulated inflammatory cells in connective tissue. No difference in gingival tissue MCPIP-1 and MALT-1 levels was observed in relation to the severity of gingival inflammation. MALT-1 levels were elevated (*p* = 0.023) with the increase in tissue *P. gingivalis* levels, and there was an association between MALT-1 and IL-8 levels (*β* = 0.054, *p* = 0.001).

**Conclusions:**

Interactions of MALT-1 levels with gingival tissue *P. gingivalis* counts and IL-8 levels suggest that activation of MALT-1 can take part in *P. gingivalis*-regulated host immune responses.

**Clinical relevance:**

Pharmacological targeting the crosstalk between immune response and MCPIP-1/MALT-1 may have benefits in periodontal treatment.

## Introduction

Post-transcriptional processes, including RNA cleavage, regulate the activation and resolution of the immune system [1, 2]. Monocyte chemotactic protein-1-induced protein-1 (MCPIP-1/Regnase-1), which was originally detected in MCP-1-treated human peripheral blood monocytes through microarrays, is a ribonuclease (RNase) and negatively regulates inflammatory responses [3, 4]. Human MCPIP-1 is composed of 599 amino acids that encode a 65.8-kDa protein, and it is composed of an ubiquitin-associated domain, proline-rich region, a PilT N-terminus like (PIN) domain followed by a CCCH-type zinc-finger (ZF) domain and a C-terminal domain [4, 5]. MCPIP-1 as a RNase participates in the post-transcriptional regulation of pro-inflammatory cytokines, such as interleukin (IL)-1β, IL-6, and IL-8 [6–8]. Furthermore, MCPIP-1 drives the resolution process of inflammation by suppression of nuclear factor kappa B (NFkB) activation, stimulation of the damaged cell clearance via apoptosis, and promoting anti-inflammatory macrophage phenotype polarization [2]. It has also a negative feedback system that reduces Regnase-1 mRNA expression in the presence of high levels of Regnase-1 protein [1]. The significance of MCPIP-1 in the immune system has been proved by genetic knockout mouse models. MCPIP-1 knockout mice, myeloid-specific MCPIP-1 knockout mice, and CD4+T cells–specific MCPIP-1 knockout mice models develop spontaneous inflammatory syndromes characterized by infiltration of immune cells to various organs as well as the production of autoantibodies [7, 9, 10].

Another essential component of both innate and adaptive immunity is the intracellular signaling protein known as mucosa-associated lymphoid tissue lymphoma translocation protein 1 (MALT-1) [11, 12]. MALT-1 acts as a scaffold protein providing a platform for assembly of other NF-κB signaling proteins in lymphoid cells, myeloid mast cells, and non-immune cells. Besides, MALT-1 has protease activity, and one of the substrates of MALT-1 is MCPIP-1 [11, 12]. MALT-1-dependent cleavage of MCPIP-1 lowers MCPIP-1 level in activated T cells and thereby stabilizes a set of pro-inflammatory cytokines identified as targets of MCPIP-1 [10, 12, 13].

Periodontal disease is an inflammatory disease of the teeth-supporting tissues, and it develops in response to imbalanced interaction between the local microbial community and the inflammatory response of the host [14, 15]. The inflammatory reaction is initiated by the recognition of pathogen-associated molecular patterns (PAMPs) by specialized pattern recognition receptors (PRRs) on host cells [16]. Recognition of PAMPs by innate immune cells stimulates the secretion of pro-inflammatory cytokines and chemokines. In the context of periodontitis, however, little is known about the expression profile of MCPIP-1 and MALT-1 in the gingival tissues in relation to the extension of infection and inflammation. Our group has demonstrated that levels of MCPIP-1 and MALT-1 are regulated by periodontitis-associated bacteria in monolayers of gingival keratinocytes [17]. It was also demonstrated that MCPIP-1 is downregulated by *P. gingivalis* in gingival keratinocytes. Moreover, it is postulated that *P. gingivalis* alter the innate immune response of gingival keratinocyte through overactivation of the NF-κB signaling pathway by *P. gingivalis*-induced degradation of MCPIP-1 [18]. Both studies indicate an interaction between these pro-inflammatory response regulatory proteins and periodontitis-associated pathogens.

In the present study, we hypothesized that the suppressed levels of MCPIP-1 and elevated levels of MALT-1 in human gingival tissues are related to the extension of infection (*P. gingivalis* colonization) and severity of clinical inflammation. Therefore, the aims of this study were to (1) localize MCPIP-1 and MALT-1 protein expression in gingival tissues and (2) profile their protein expression in relation to the clinical inflammation, *P. gingivalis* colonization, and IL-8 levels.

## Materials and methods

### Study populations and tissue sampling

Altogether, 57 study samples were collected from two independent populations: (1) Sixteen gingival tissues were collected from periodontally healthy individuals and from periodontitis patients to localize MCPIP-1 and MALT-1 immunohistochemically, and (2) forty-one gingival tissue samples were collected from 20 periodontitis patients to evaluate the MCPIP-1 and MALT-1 tissue levels in relation to clinical inflammation, gingival tissue *P. gingivalis* counts and gingival tissue IL-8 levels. None of the participants were compensated financially, but all participants received free periodontal treatment. Detailed information on population characteristics and sample collection procedures are given below.

### Study population for the localization of MCPIP-1 and MALT-1 in gingival tissues

Eight periodontitis patients (5 females and 3 males with the mean age of 36.8 ± 3.5 years) and eight periodontally healthy individuals (6 females and 2 males with the mean age of 35.8 ± 3.2) were recruited for this study. All participants were systemically healthy and non-smokers. None of the participants took any systemic or local medications (antibiotics, analgesics, etc.) 6 months prior to collecting gingival tissue samples. Individuals with active oral mucosal lesions, caries, ongoing orthodontic treatment, or pregnancy and lactation were excluded from the study. The study protocol was approved by the University of Istanbul’s Faculty of Dentistry’s Ethics Committee in accordance with the Helsinki Declaration (2017/41), and the study was conducted in accordance with the Helsinki Declaration of 1975, as revised in 2013. All participants gave written informed consent. A single calibrated clinician (G.K.) carried out the clinical examinations. Plaque index (PI), bleeding on probing (BOP), probing pocket depth (PPD), and clinical attachment level (CAL) were measured from each tooth by using a periodontal probe (UNC-15, Hu-Friedy, Chicago, IL, USA), and the index scores were recorded. Radiographic bone loss was evaluated to confirm the periodontitis diagnosis. In all periodontitis patients, the percentage of sites with PPD ≥ 5 mm, CAL > 5 mm, and BOP positivity were over 30%. Moreover, excessive accumulation of microbial dental plaque and calculus were visible. Based on the staging and grading of the disease, periodontitis of recruited periodontitis patients for this study was defined as Stage III, Grade B [19]. Individuals with no clinical symptoms of gingival inflammation and no sites with PPD > 3 mm were considered periodontally healthy [20].

### Gingival tissue sample collection for the localization of MCPIP-1 and MALT-1

Inflamed gingival tissue samples were collected from the deepest mesial/distal periodontal pockets of the intact adjacent teeth of periodontitis patients during periodontal flap surgeries. Tissue samples with no signs of inflammation were harvested during crown lengthening procedures or orthodontic tooth extractions from periodontally healthy individuals. Internal beveled incision by using a carbon steel surgical scalpel blade was a method of choice for gingival tissue sample collection. This method allowed us to reach to the bottom of the periodontal pocket and sulcular epithelium. With this, it was possible to focus on pocket/sulcular epithelium and underlying connective tissue as region of interest, instead of collecting oral epithelium, which is far from the zone of infection. All tissue biopsies were performed between 21 May 2015 and 27 February 2016 at the periodontology clinics of Istanbul University Faculty of Dentistry, Istanbul, Turkey. Immediately after the harvest of the gingival tissue, the oral epithelium was marked with a tissue marking dye (CDI’s tissue marking dye, 0724-2, Cancer Diagnostics Inc., Dunham, NC, USA) to facilitate the orientation while embedding biopsies in paraffin blocks and the recognition of the sulcular/oral epithelium sites of the biopsies during the microscopic evaluation. All specimens were fixed in 4% formalin solution overnight (16–20 h) and embedded in paraffin blocks. Immunohistochemical analysis was performed in the University of Turku, Institute of Dentistry laboratories.

### Immunohistochemical localization of gingival tissue MCPIP-1 and MALT-1

Paraffin-embedded gingival tissue samples were immunostained with hematoxylin and eosin, thymidine blue, primary antibodies against MCPIP-1 (1:200 dilution, Cat # PA5-24458, Thermofisher, USA) and against MALT-1 (1:100 dilution, Cat# PA5-79622, Thermofisher, China) using an automated immunostainer. The primary antibodies were detected with goat anti-Rabbit IgG secondary antibody (1:10000 dilution, Cat#31460, Thermofisher, USA) and streptavidin–horseradish peroxidase and visualized with 3,3′ diaminobenzidine tetrahydrochloride in horseradish peroxidase buffer. Human lung (for MALT-1) and human kidney (for MCPIP-1) tissues were used as positive staining controls. Extra sets of staining omitting primary antibodies were performed as negative controls. The immunohistochemical stainings were photographed using a light microscope (Leica DMLB, Leica, Wetzlar, Germany).

### Study population for the determination of MCPIP-1, MALT-1, *P. gingivalis*, and IL-8 gingival tissue levels

Twenty (12 males and 8 females with an age range of 24–70 years) periodontitis patients diagnosed according to the 2017 World Workshop for classification of periodontal and peri-implant diseases and conditions [19], referred to the Hacettepe University, Faculty of Dentistry Department of Periodontology, were recruited for the study. The Ethical Committee of Hacettepe University reviewed and approved the study protocol (No: GO 17/786-34). All participants gave written informed consent, and the study was conducted in accordance with the Helsinki Declaration of 1975, as revised in 2013. Exclusion criteria were systemic diseases, pregnancy, lactation, smoking, use of antibiotics and/or anti-inflammatory drugs within 3 months, and periodontal treatment within 6 months preceding the study. A single calibrated examiner (M.Ö.K.) performed a full periodontal examination. PPD, modified gingival index (MGI), CAL, and PI were measured by using a manual periodontal probe (UNC-15, Hu-Friedy, Chicago, IL, USA). A total of 41 periodontal pocket sites with 5–7 mm PPD were included in the study. Sampling sites were divided into three study groups based on the level of inflammation:

(i) Marginal inflammation group: Slight changes in color and texture but not in all portions of gingival margin or papilla (*n* = 11, mean PPD: 5.18 ± 0.6, mean MGI: 1 ± 0, mean CAL: 6.09 ± 1.21, mean PI: 1.27 ± 0.47),
(ii) Mild inflammation group: Slight changes in color and texture in all portions of gingival margin or papilla (*n* = 14, mean PPD: 5.43 ± 0.85, mean MGI: 2 ± 0, mean CAL: 6.21 ± 1.25, mean PI: 1.71 ± 0.47),
(iii) Moderate to severe inflammation group: Bright surface, erythema, edema, ulceration, or spontaneous bleeding tendency (*n* = 16, mean PPD: 5.13 ± 0.34, mean MGI: 3.26 ± 0.45, mean CAL: 6.44 ± 0.96, mean PI: 2.31 ± 0.48).

Samples were collected between December 2017 and March 2018 at Hacettepe University, Faculty of Dentistry, Department of Periodontology, Turkey.

### Gingival granulation tissue sample collection for the determination of MCPIP-1, MALT-1, *P. gingivalis*, and IL-8 levels

Inflammatory gingival granulation tissues were excised by curettes (Gracey curettes, Hu-Friedy, Chicago, IL, USA) as previously described [21]. Granulation tissue biopsies were stored at −80 °C and transferred to the Institute of Dentistry, University of Turku, Finland, for the analyses.

Samples were prepared for analysis at the University of Turku, Institute of Dentistry, in June 2018. Tissue samples were cut into small pieces, lysed in a 500 μl solution of 50 mM Tris-Cl, 150 mM NaCl and 1% Triton X-100, vortexed for 10 s, and incubated for 24 h at 4 °C. Lysates were then centrifuged at 10,000 g for 1 min. The obtained supernatants were aliquoted for determinations of MCPIP-1, MALT-1, *P. gingivalis*, and IL-8 concentrations.

### Determination of gingival tissue MCPIP-1 and MALT-1 levels with immunoblots

The total protein level of each gingival tissue sample was determined by a commercial protein assay (Bradford, Bio-Rad, Hercules, CA, USA). Samples containing equal amounts of protein (10 μg/ml, 144 ng) were resolved by 10% sodium dodecyl sulfate (SDS)-polyacrylamide gel electrophoresis, and proteins were transferred to polyvinylidene difluoride (PVDF) membranes (Trans-Blot Turbo Transfer System, Bio-Rad, Hercules, CA, USA). Membranes were incubated with primary antibodies against MCPIP-1 (1:750 dilution, Cat#PA5-24458, Thermofisher, USA) and MALT-1 (1:1000 dilution, Cat#PA5-79622, Thermofisher, China) at 4 °C. Afterwards, the membranes were incubated with Goat anti-Rabbit IgG (H+L) secondary Antibody (1:10000 dilution, Cat#31460, Thermofisher, USA). The horseradish peroxidase was detected by a commercial chemiluminescent substrate reagent kit (Novex ECL, Invitrogen, Carlsbad, CA, USA). A blot imaging system (ChemiDoc, MP Imaging System, Bio-Rad, Hercules, CA, USA) was used to detect the bands on the membranes. The bands were quantified using a software (ImageJ, National Institute of Health, Bethesda, MD, USA).

### Determination of gingival tissue *P. gingivalis* counts

Detailed descriptions of *P. gingivalis* tissue level determination methods were published previously [21]. Briefly, 100 μl of gingival granulation tissue sample was mixed with proteinase K in bead tubes. Tubes were agitated (Mixer Mill MM301, Retch GMBH, Haan, Germany) and centrifuged. The DNA was extracted with the phenol–chloroform method. *P. gingivalis* counts in gingival granulation tissue were analyzed using a quantitative single-copy gene-based real-time polymerase chain reaction (qPCR) technique [22]. Analyses of qPCR were performed with the following steps: initial denaturation at 95 °C for 3 min, followed by 40 cycles of 3 s at 95 °C and 20 s at 60 °C. *P. gingivalis* DNA samples were used as positive controls and water as a negative control. The results are presented as genomic copies/ng DNA. The detection limit for *P. gingivalis* was 23 genome equivalents.

### Determination of gingival tissue *P. gingivalis* gingipain activity

*P. gingivalis* gingipain activity was determined using gingipain-specific fluorogenic substrate [FITC-Ahx-(L)Arg-(D)Arg-KDbc (RR)], as previously described [21]. Briefly, a 16 μM of gingipain substrate was added in 50 μl of tissue supernatant and supplemented with 2.5 μM of L-cysteine. The fluorescence activity was read at 37 °C for 1 h with 2-min intervals on a fluorescence microplate reader (Ex:485 nm–Em: 530 nm, Biotek Instruments) and was defined in relative fluorescence activity per min (RF/min). All enzyme tests were performed in triplicate.

### Determination of gingival tissue IL-8 levels

Tissue concentrations of IL-8 were detected by a bead-based multiplexed immunoassay system (Luminex xMAP, Bio-Rad, Hercules, CA, USA) with the commercial kits (Bio-Plex Pro Human Inflammation Panel 1, Bio-rad, Hercules, CA, USA) according to the manufacturer’s instructions. The limit of detection of the assay was 2.7 pg/ml for IL-8.

### Statistical analysis

Data distributions were analyzed with Shapiro–Wilk test. Non-parametric Kruskal–Wallis (for multiple comparisons) and Mann–Whitney *U* tests were used in between-group comparisons, and *p* < 0.05 was accepted as statistically significant. Linear regression analysis was used to determine the unadjusted and adjusted (level of inflammation) associations of IL-8 with tissue levels of MCPIP-1, MALT-1, *P. gingivalis* counts, and *P. gingivalis* gingipain activity. A commercial software (IBM SPSS V26.0, IBM, Armonk, NY, USA) was used for statistical analyses.

## Results

**Fig. 1:**
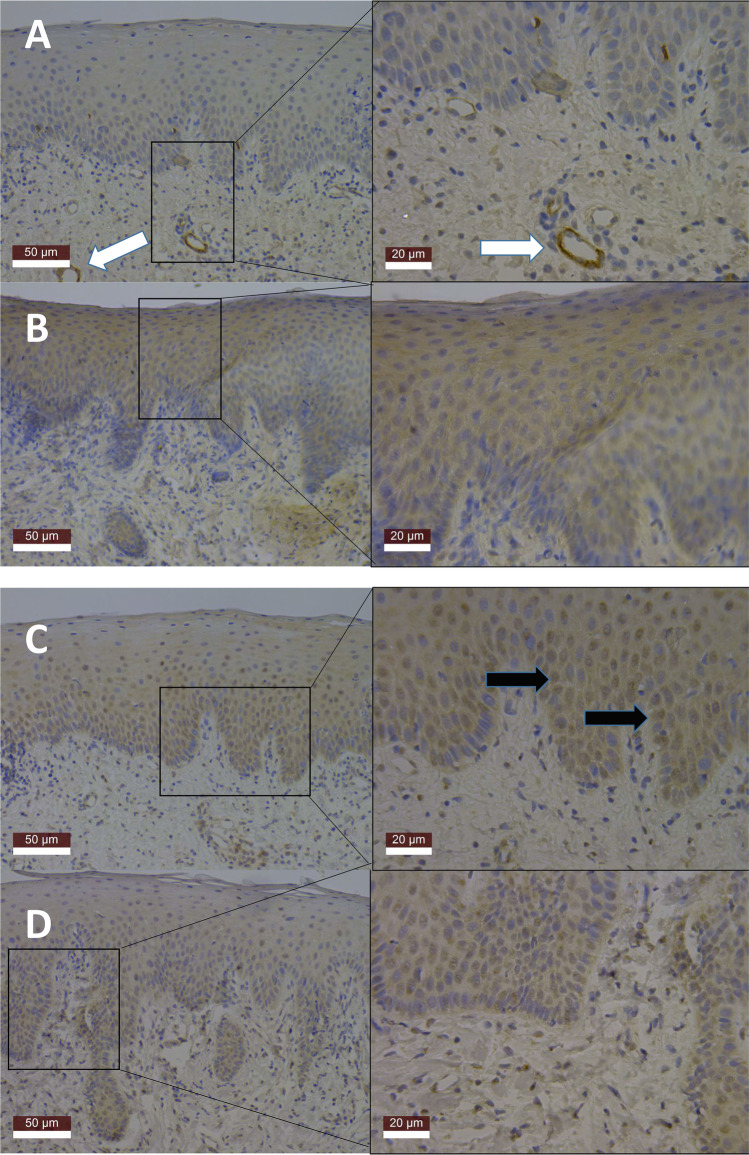
Expression and localization profiles of MCPIP-1 (**A**, **B**) and MALT-1 (**C**, **D**) in human gingival tissues with low (20X, left column) and high (40X, right column) magnifications. Images (**A**) and (**C**) are from periodontally healthy tissues and (**B**) and (**D**) from periodontitis tissues. White arrows indicate MCPIP-1-stained blood vessel walls, and black arrows indicate positively stained cells for MALT-1

Immunohistochemical analyses revealed that MCPIP-1 was detectable in epithelium and in connective tissues. MCPIP-1 was prominent especially around the blood vessel walls in healthy gingival tissues and in the epithelium in periodontitis-affected tissues. MCPIP-1 stained cells were more evident in periodontitis-affected tissues than in healthy periodontal tissues, accumulated around the layers of stratum spinosum and stratum granulosum of gingival epithelium, and less observed in stratum basale and stratum corneum (Fig. 1A, B). In both healthy and periodontitis-affected human gingival tissues, MALT-1 was detectable at all layers of gingival epithelium being especially prominent at the layers of stratum basale, stratum spinosum, and stratum granulosum and less observed in stratum corneum (Fig. 1C, D). MALT-1 was also observed around the accumulated inflammatory cells of connective tissue.

**Fig. 2:**
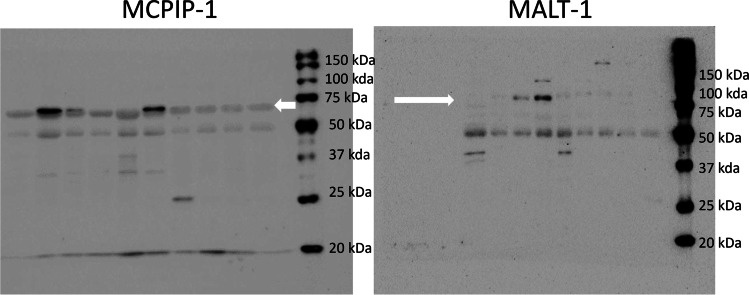
Tissue MCPIP-1 and MALT-1 protein profiles according to immunoblots. MCPIP-1 was observed at 70, 50, ~37, and 25 kDa, while MALT-1 was detected at 90, 50, and ~45 kDa. Each lane belongs to one gingival tissue sample. White arrows indicate the band closest to the expected kDa range

**Fig. 3:**
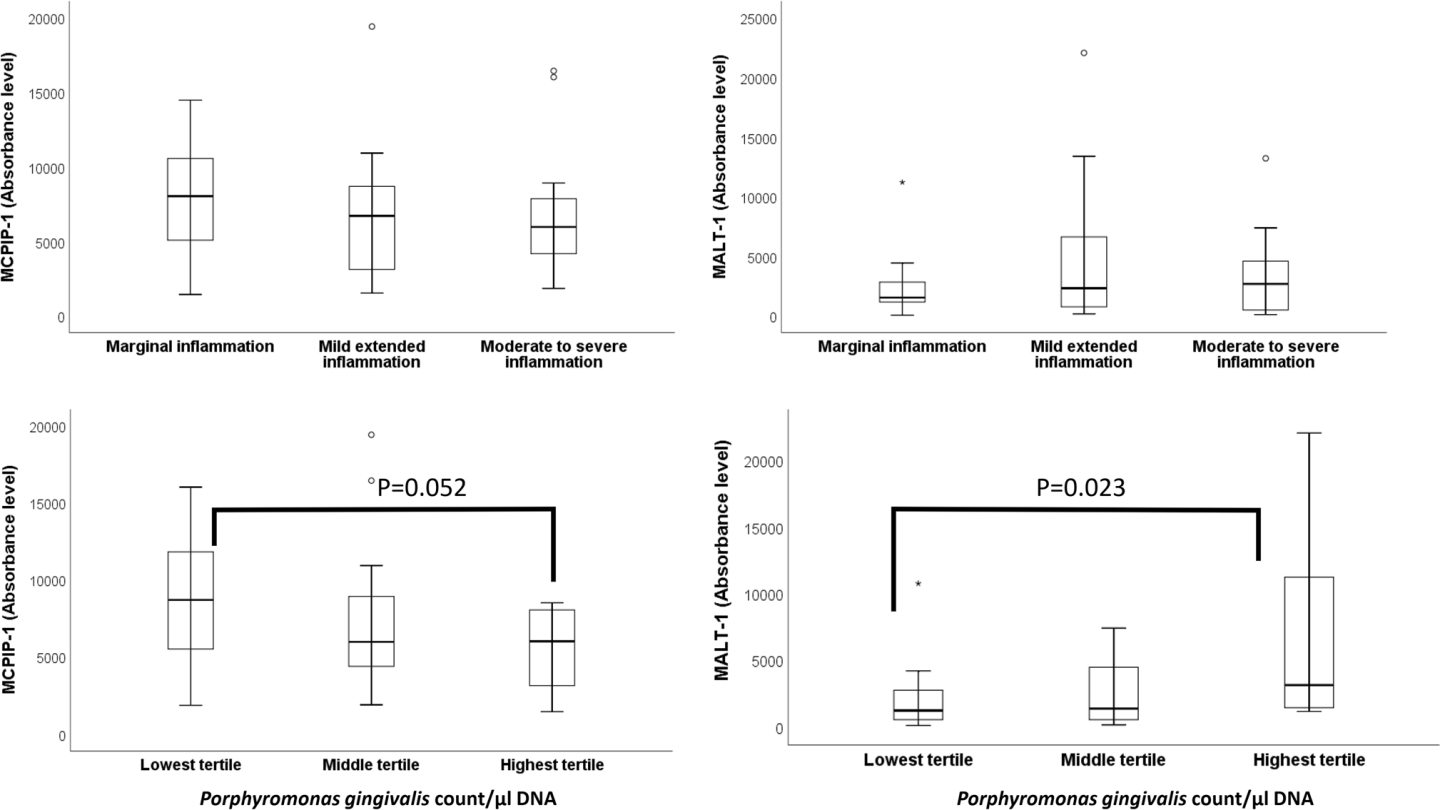
Human gingival tissue MCPIP-1 and MALT-1 levels according to the level of gingival inflammation and gingival tissue *Porphyromonas gingivalis* levels. Statistical differences between the groups are demonstrated with connecter lines and *p* values

MALT-1 and MCPIP-1 were detected in all gingival granulation tissues by immunoblots, while IL-8 could not be detected with the Luminex assay in 8 samples. In immunoblot analyses, MCPIP-1 (expected molecular weight 62 kDa) was detected at ~65, 50, 37, and 25 kDa, while MALT-1 (expected molecular weight 92 kDa) was detected at ~90, 50, and 45 kDa (Fig. 2). The difference in the gingival granulation tissue MCPIP-1, MALT-1, *P. gingivalis*, *P. gingivalis* gingipain activity, and IL-8 levels in relation to gingival inflammation was not statistically significant among the marginal, mild, and moderate to severe inflammation groups (Table 1, Fig. 1). MCPIP-1 levels tended to decrease (*p* = 0.052) and MALT-1 levels elevated (*p* = 0.023) with the increase in tissue *P. gingivalis* counts (Fig. 3). Linear regression analysis indicated a significant association between MALT-1 and IL-8 levels (*p* = 0.001) after being adjusted for level of gingival inflammation. No other significant association was observed between granulation tissue IL-8 levels and MCPIP-1 levels, *P. gingivalis* counts, or *P. gingivalis* gingipain activity (Table 2).

**Table 1:** Tissue levels of MCPIP-1, MALT-1, *Porphyromonas gingivalis* counts, *P. gingivalis* gingipain activity, and IL-8. Data is presented as median (25th–75th percentile). No statistical difference was observed between the inflammation groups

Level of inflammation	MCPIP-1 (absorbance level)	MALT-1 (absorbance level)	*P. gingivalis* (count/μl DNA)	*P. gingivalis* gingipain activity [relative fluorescence per minute (RF/min)]	IL-8 (pg/μg protein)
Marginal inflammation	8040 (4491–11,809)	1544 (1160–3803)	676 (0–5696)	0.83 (0.33–4.8)	11.4 (8.68–13.9)
Mild inflammation	6714 (3053–8926)	2331 (704–7658)	1123 (19.5–4054)	1.1 (0.8–1.08)	5.8 (3.45–18.6)
Moderate to severe inflammation	5972 (4084–8346)	2693 (506–4808)	391 (20–723)	2.4 (1.04–5.6)	12.8 (9.6–24.5)

**Table 2:** Unadjusted and adjusted (level of inflammation) associations of tissue IL-8 levels with tissue levels of MCPIP-1, MALT-1, *Porphyromonas gingivalis* counts, and *P. gingivalis* gingipain activity

	Unadjusted (*β*, 95% CI, *p*)	Adjusted (*β*, 95% CI, *p*)
MALT-1	0.579, 0.001–0.003, < 0.001	0.054, 0.001–0.003, 0.001
MCPIP-1	−0.006, −0.001–0.001, 0.975	0.003, −0.001–0.001, 0.985
*Porphyromonas gingivalis* tissue counts	0.229, −0.001–0.003, 0.199	0.257, −0.001–0.004, 0.165
*P. gingivalis* gingipain activity	0.314, −0.049–0.948, 0.076	0.310, −0.069–0.959, 0.087

## Discussion

To our knowledge, our study is the first to demonstrate the presence of MCPIP-1 and MALT-1 in gingival tissues and reveal interactions between gingival tissue MALT-1, *P. gingivalis*, and IL-8 levels. According to our results, the expression profile of MALT-1, which is a suppressor of pro-inflammatory response inhibitors, is related to tissue *P. gingivalis* levels and associated with IL-8 levels. *P. gingivalis* is known to carry various virulence mechanisms that can suppress or activate immune-response components. By stimulating MALT-1 expression, *P. gingivalis* may suppress anti-inflammatory mechanisms and induce constant and uncontrolled inflammation.

In the present study, both immunohistochemical and immunoblotting techniques were used to identify MCPIP-1 and MALT-1 in gingival tissues. It is known that the western blot provides a relative comparison of protein levels, as the detected signals are not linear across the concentration range of samples. From the molecular biology perspective, however, as a common mechanism of many proteins, MCPIP-1 also goes through some post-translational modifications, such as phosphorylation and ubiquitation. The primary antibodies used in western blot can not target specifically those modification residues that may lead to a limitation on the signal detection. In addition, creating study groups with variations in the severity of inflammation allowed us to demonstrate the regulation of MCPIP-1 and MALT-1 protein expression in relation to clinical inflammatory status. A relatively small sample size was a limitation of this study. Another limitation was that the mRNA expression profiles of MALT-1, MCPIP-1, and IL-8 were not included in the study design, as the amount of tissue samples was not enough to expand the study aims. Future studies may also consider implementing chronic gingivitis groups to their study designs, as these cases may carry strong suppressive mechanisms against pro-inflammatory responses, including MCPIP-1. A final limitation is the lack of subcellular localization of MCPIP-1 and MALT-1. Subcellular localization of proteins is beneficial in inferring the function of them [23]. Even though the directly observation of the fluorescent-labeled protein with microscopic imaging in situ and improving the outcome data with machine learning techniques is one common technique, the amount of tissue samples was not enough to expand the study aims.

In our study, tissue MCPIP-1 levels tend to decrease (*p* = 0.052) with the increase in tissue *P. gingivalis* levels. As one of the major periodontal pathogens, *P. gingivalis* interacts with the immune system through its virulence factors including gingipains, lipopolysaccharides (LPS), and fimbriae [24]. *P. gingivalis* gingipains regulate the expression profiles of inflammatory cytokines and degrade antimicrobial peptides post-transcriptionally [24]. Recognition of *P. gingivalis* LPS by toll-like receptors activates NF-κB and mitogen-activated protein kinase (MAPK) signaling pathways [25]. It was found that LPS-mediated activation of inhibitor of transcription factor NF-kB kinase (IKK) complex phosphorylates MCPIP-1 rapidly and then phosphorylated MCPIP-1 undergoes ubiquitination and rapid degradation [26]. Moreover, it was demonstrated that *P. gingivalis* gingipain activity leads to a rapid degradation of MCPIP-1 in gingival keratinocytes, [17] which contributes the inflammophilic pathobiont formation [18]. In contrast to the observations, it was shown that lipopolysaccharide treatment increases the expression of MCPIP-1 through the activation of TLR-4 in murine macrophage cell line Raw264.7, in mouse primary bone marrow-derived macrophages, and in human monocyte-derived macrophages [27, 28]. One can suppose that in gingival tissue exposed to *P. gingivalis*, the MCPIP-1 expression decreases rapidly among others through LPS-mediated activation of IKK, which induced ubiquitin-proteasome-mediated degradation and also via the degradation by gingipains, but the expression of MCPIP-1 increases over time among others through the activation of TLR-4. Previous studies have focused on the activation of MCPIP-1 as part of the immediate cellular response against pro-inflammatory stimulants, but little is known about tissue MCPIP-1 response in relation to pathogen colonization. Gasiorek [18] (2021) and coworkers detected that *P. gingivalis* induced a significant decrease in MCPIP-1 protein levels in murine gingiva. Moreover, by visualizing the MCPIP-1 protein level with a confocal laser scanning microscope, they showed that a decrease of MCPIP-1 immunostaining intensity coincided with gingipain invasion into the cytoplasm. Our results are in line with Gasiorek [18] et al. (2021) and demonstrate that increase in tissue *P. gingivalis* levels suppresses MCPIP-1 levels. Our findings are relatively new and require validation in animal and human studies before it can be claimed that suppression of MCPIP-1 in gingival tissues by *P. gingivalis* is part of periodontitis pathogenesis.

The results of this study demonstrate that MALT-1 tissue levels get elevated with the increase in *P. gingivalis* counts in gingiva. It was shown that in macrophages, LPS triggers a pro-inflammatory signal, which in turn activates MALT-1/BCL10/CARD9 signalosome formation and then MCPIP-1 cleavage [9]. Indeed, by inhibiting the phosphorylation of MAPK and NF-κB activation via its deubiquitinase activity, MCPIP-1 contributes to LPS hyporesponsiveness induced by subsequent LPS stimulation and macrophage reprogramming [29]. Gasiorek [18] (2021) and coworkers detected an increased activity of MALT-1 in keratinocytes at 4 h and 24 h post *P. gingivalis* infection. Moreover, it was demonstrated that the cleavage of MCPIP-1 by MALT-1 in T helper cells is induced in response to antigen reaction [10]. Based on these findings, it is possible to propose that *P. gingivalis* suppress the anti-inflammatory MCPIP-1 function by stimulating the MALT-1 expression to maintain a chronic and uncontrolled inflammatory stage. However, as the design of this study is cross-sectional, we cannot prove a cause-effect relationship.

In the present study, we observed an association between the tissue levels of MALT-1 and IL-8, but not between MCPIP-1 and IL-8. MALT-1 is essential for NF-κB activation [11, 12]. It is reasonable to postulate that MALT-1 increases IL-8 levels by reinforcing the activation of NF-κB signaling induced by PAMPs. Dobosz [6] (2016) and coworkers found that MCPIP-1 expression in epithelial cells decreases the transcription and translation of IL-8. Whereas in the present study, no statistically significant relationship was observed between levels of IL-8 and MCPIP-1 in gingival granulation tissues. Indeed, it is demonstrated that the level of IL-8 in gingival tissues is regulated by various mechanisms [30–32], and degradation of IL-8 mRNA by MCPIP-1 is one of them. Animal studies with MCPIP-1 knockout models may clarify the relationship between gingival IL-8 and MCPIP-1 levels.

Periodontal health requires a delicate balance between the commensal oral microbiota and the host. The dental biofilm maintains the health of gingival tissue by contributing to the turnover and homeostasis of the oral tissues [33–35]. The findings of the present study shed light on the interactions between periodontal infection-induced inflammation and MCPIP-1/MALT-1 axis. Further studies should be conducted to elucidate the role of MCPIP-1/MALT-1 in periodontitis and determine whether targeting the crosstalk between immune response and MCPIP-1/MALT-1 has benefits in periodontal treatment. Finally, smoking, which is a major risk factor for periodontitis, may influence the expressions of MCPIP-1 or MALT-1 through its effects on immune cell response or oral microbial colonization and thus must be taken into account in further studies.

## Conclusion

In conclusion, present findings indicate a relation between gingival MALT-1 levels with gingival tissue *P. gingivalis* counts and IL-8 levels. Further studies on the interactions between tissue levels of *P. gingivalis*, pro-inflammatory cytokine RNase MCPIP-1, and its inhibitor MALT-1 shed light to new mechanisms by which *P. gingivalis* activate or suppress host immune response.

## Data Availability

It is possible to access study data and materials from the authors by permission.
